# The Moorfields Safer Surgery System

**DOI:** 10.4103/0974-9233.56220

**Published:** 2009

**Authors:** Sumit Dhingra, Peng T. Khaw

**Affiliations:** From Ocular Repair and Regeneration Biology Research Unit, NIHR Biomedical Research Centre, Moorfields Eye Hospital and UCL Institute of Ophthalmology, London, United Kingdom

**Keywords:** Glaucoma, trabeculectomy, surgery

## Abstract

This review presents the ‘Moorfields Safer Surgery System’, which is designed to improve the consistency and outcomes of trabeculectomy surgery. Evidence-based recommendations are made for each step of the surgery. This system requires a minimum of equipment and can be easily implemented by most surgeons. The system is ultimately designed to preserve the vision in our patients by minimising complications while maintaining a desired intraocular pressure.

## INTRODUCTION

The Moorfields Safer Surgery System is designed to improve surgical outcomes for our patients following trabeculectomy. Our fundamental observation that cystic blebs have restricted posterior flow with the so-called “ring of steel” was critical in the initial conception of this system. Over the years the system has evolved to include many other features based on evidence from basic science and clinical research. A comprehensive description of the system is available elsewhere;^1^ below is a less detailed summary of our recommendations.

## SITE FOR SURGERY

The superior half of the globe is the safest site for the trabeculectomy due to the reduced incidence of inflammation, endophthalmitis, dysthesia and recurrent subconjunctival haemorrhages.^2^–^4^ The upper lid can also provide mechanical protection and covers the iridectomy, preventing diplopia. In cases where the only sites available are interpalpebral or inferior, we suggest that a tube drainage device should be preferred.

## TRACTION SUTURE

**Figure 1:**
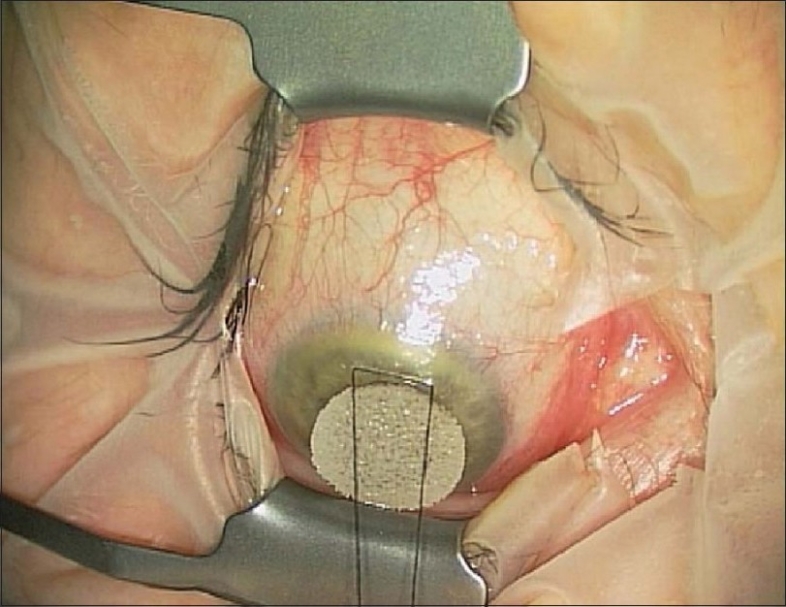
Corneal traction suture

We prefer a corneal traction suture (7/0 black silk suture on a semi-circular needle) to a superior rectus suture [Figure 1]. This removes the possibility of a superior-rectus haematoma with the added benefits of a superior vector force^5^ and a reduced risk of failure.^6^ When the suture is placed at the correct depth, the risk of either ocular penetration or alternatively “cheese-wiring” and loss of traction is minimised.

## CONJUNCTIVAL INCISION

In the past we made a conjunctival incision deep in the fornix (limbus based conjunctival flap) to reduce the chance of a wound leakage. Although, with this incision, a diffuse bleb can be achieved, a limbal conjunctival incision (fornix based conjunctival flap) is a less technically difficult method to achieve posterior aqueous and a diffuse bleb.^7^–^9^ To minimise surgical trauma and wound leakage, a conjunctival incision of 5-10 mm is made at the limbus without any relieving incisions.

## SCLERAL FLAP

We create a rectangular scleral flap by first cutting a horizontal incision parallel to the limbus, then dissecting a partial thickness scleral pocket (much like a phacoemulsification pocket) and finally cutting the two side incisions. By not completing the side incisions right up to the limbus, the aqueous is directed backwards over a wider area, encouraging greater posterior flow and a more diffuse bleb.

The scleral flap must be large enough to provide resistance and be not too thin so as to risk dehiscence or “cheese-wiring” of the flap sutures. Any large aqueous veins at the potential site of the flap must be avoided.

## CONJUNCTIVAL POCKET

A wide pocket for applying the antimetabolite sponges behind the flap and underneath the conjunctiva is made with Westcott scissors. When dissecting over the superior rectus, care must be taken to lift the conjunctiva and to cut attachments avoiding the tendon. Any intra-operative antimetabolites should be applied after constructing the scleral flap but before the eye is entered, so if there are any concerns, the antimetabolites can be withheld.

## ANTIMETABOLITE TREATMENT DURATION

A large surface area is treated with antimetabolite to reduce the development of a ring of scar tissue. Based on our pharmacokinetic experiments, mitomycin C (MMC) is applied at a concentration of 0.2 or 0.5 mg/ml for three minutes, or alternatively 5-Fluorouracil (5-FU) 50 mg/ml may be used.^10^,^11^ The antimetabolite must then be washed out thoroughly, with at least 20 ml of balanced salt solution.

## SPONGES AND CONJUNCTIVAL CLAMP

**Figure 2:**
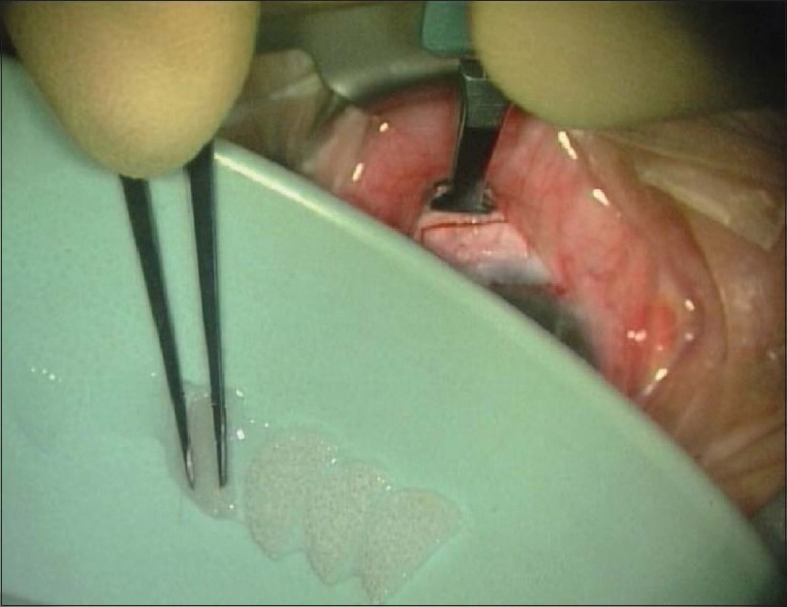
Non-fragmenting, circular medical grade polyvinyl alcohol sponges cut in half

We use approximately three circular medical grade polyvinyl alcohol sponges (which are usually used for LASIK as corneal shields) as they do not fragment.^12^ These are either cut in half or whole, and folded like a lens to pass comfortably into the conjunctival pocket without making contact with the edges, reducing the chances of postoperative wound leakage or dehiscence [Figure 2]. For the same reason, we also use a custom made clamp designed to hold back the conjunctiva (Khaw Small Conjunctival Clamp No.2-686; Duckworth and Kent Ltd.) to insert and remove the sponges.

## PARACENTESIS AND INFUSION

A paracentesis allows improved control of the anterior chamber depth. If it is made obliquely and parallel to the limbus, it reduces the possibility of lens damage. Furthermore, if it is also sited inferiorly it may also be used post-operatively in the clinic. For enhanced pressure control, we use a continuous anterior segment infusion (Lewicky; Visitec Company, Sarasota, FL), which continuously maintains the pressure, with the flexibility to adjust it by changing the bottle height.

## SCLEROSTOMY

Although a sclerostomy can be made manually with a blade and scissors, we favour a specially designed punch (Khaw Small Descement Membrane Punch No. 7-101; Duckworth and Kent Ltd). This allows superior anterior access (even if the sides of the sclerosotomy are not fully exposed) and permits the relatively straightforward construction of a small sclerostomy, slightly larger than the head of the punch (0.5 mm diameter). This size is functionally adequate, as well as having the benefit of reduced astigmatism and reduced anterior aqueous flow.^13^ A perpendicular, clean, non-shelved incision should be made precisely at the sclerolimbal junction. This will then appear at the anterior part of the trabecular meshwork reducing the chance of bleeding or damage to the ciliary body.

## PERIPHERAL IRIDECTOMY

A peripheral iridectomy prevents iris incarceration in the sclerostomy, therefore relieving any element of pupillary block. By cutting it with scissors parallel to the limbus, a relatively small defect can be made, reducing the chances of glare and diplopia. As we use an infusion, gentle pressure on the back of the wound brings the iris into the wound, allowing the iridectomy to be cut without intraocular manipulation, which minimises trauma and the need for an assistant.

## SCLERAL FLAP SUTURES

The function of the sutures is to secure the flap so that it acts as an aqueous flow restrictor. This may be particularly important when antimetabolites are used, or in an eye with angle closure. We usually pre-place, but do not tie, a couple of sutures while the eye is still firm.

Once ready, we first tie the sutures at the posterior corner of the scleral flap (Alcon; 10/0 monofilament nylon suture). The need for further sutures is then assessed by the amount of aqueous flowing through the flap (by either having a continuous anterior chamber infusion or by manually inflating the eye). The advantage of an infusion is that the flow and final intra-ocular pressure can be set more accurately.

**Figure 3:**
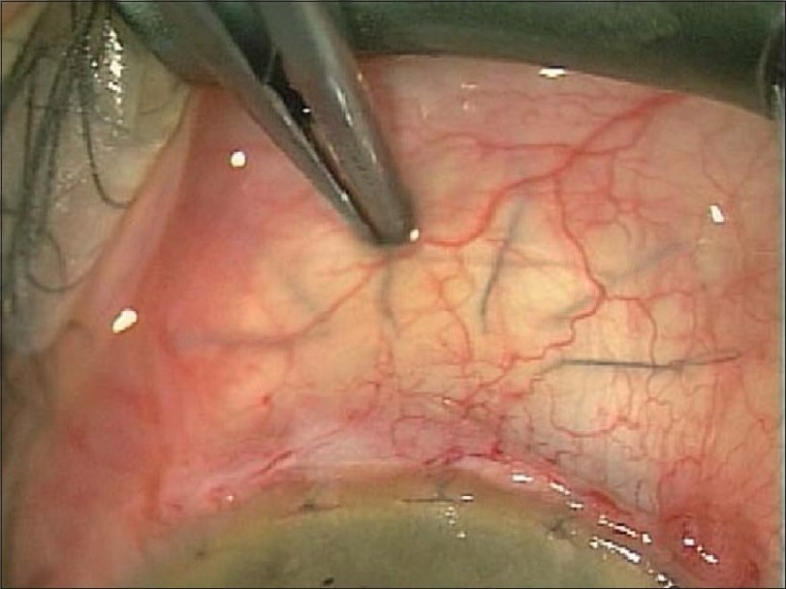
Transconjunctival adjustment of suture tension with specially designed forceps

The scleral flap sutures may need to be adjusted in the post-operative period to increase flow. This should be done cautiously as hypotony may result, even several months post-operatively. Sutures can be removed by laser suture-lysis, though there is a risk of suddenly lowering the pressure. Therefore, we prefer the safer releasable suture, or our adjustable suture technique.^14^,^15^ The new type of adjustable suture allows precise post-operative control of the suture tension through the conjunctiva with a specially designed forceps which has smooth edges (Khaw Transconjunctival Adjustable Suture Control Forceps, No. 2-502; Duckworth and Kent Ltd.,) [Figure 3].

## CONJUNCTIVAL CLOSURE

**Figure 4:**
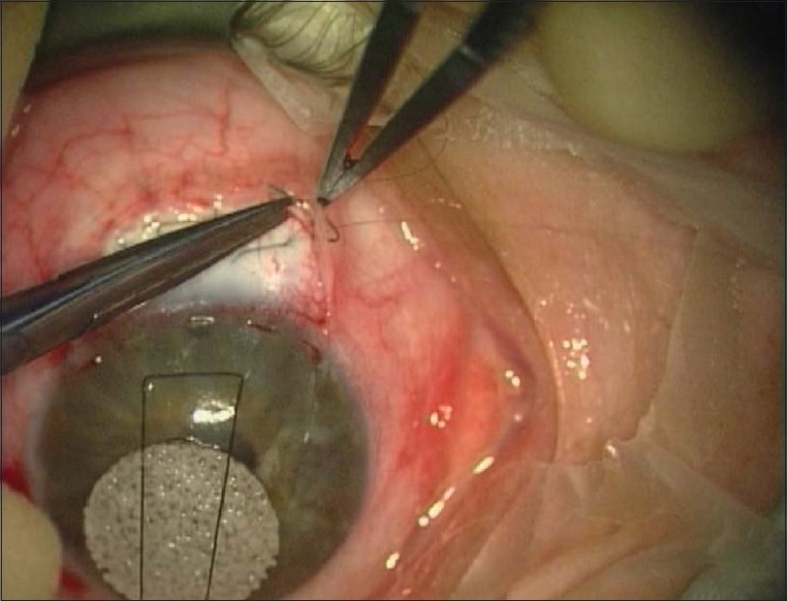
Closure of the conjunctiva with buried knots in shallow corneal grooves

When suturing conjunctiva, it is essential to use a round-bodied (rather than a spatulated) needle as any conjunctival holes will close more spontaneously. Although there are many alternatives, we prefer a more comprehensive conjunctival closure with a continuous nylon suture and buried knots in shallow corneal grooves [Figure 4]. This technique has in effect eliminated central conjunctival retraction, leaks and suture discomfort in our patients.

## CONCLUSIONS

The Moorfields Safer Surgery System has improved the surgical outcomes for our patients following trabeculectomy.^7^ We strongly believe that by following this evolving system, in combination with newer anti-scarring agents, it will be possible in the future to even further increase the efficacy and safety of glaucoma surgery.

Video downloads of the Moorfields Safer Surgery System are available at: http://www.ucl.ac.uk/ioo/research/khawlibrary
